# The challenge to detect heart transplant rejection and transplant vasculopathy non-invasively - a pilot study

**DOI:** 10.1186/1749-8090-4-43

**Published:** 2009-08-16

**Authors:** Engin Usta, Christof Burgstahler, Hermann Aebert, Stephen Schroeder, Uwe Helber, Andreas F Kopp, Gerhard Ziemer

**Affiliations:** 1 Department of Thoracic-, Cardiac- and Vascular Surgery, Tübingen University Hospital, Germany; 2 Department of Internal Medicine, Division of Cardiology, Tübingen University Hospital, Germany; 3 Department of Diagnostic Radiology, Tübingen University Hospital, Germany

## Abstract

**Background:**

Cardiac allograft rejection and vasculopathy are the main factors limiting long-term survival after heart transplantation.

In this pilot study we investigated whether non-invasive methods are beneficial to detect cardiac allograft rejection (Grade 0-3 R) and cardiac allograft vasculopathy. Thus we compared multi-slice computed tomography and magnetic resonance imaging with invasive methods like coronary angiography and left endomyocardial biopsy.

**Methods:**

10 asymptomatic long-term survivors after heart transplantation (8 male, 2 female, mean age 52.1 ± 12 years, 73 ± 11 months after transplantation) were included. In a blinded fashion, coronary angiography and multi-slice computed tomography and ventricular endomyocardial biopsy and magnetic resonance imaging were compared against each other.

**Results:**

Cardiac allograft vasculopathy and atherosclerosis were correctly detected by multi-slice computed tomography and coronary angiography with positive correlation (r = 1). Late contrast enchancement found by magnetic resonance imaging correlated positively (r = 0.92, r^2 ^= 0.85, p < 0.05) with the histological diagnosis of transplant rejection revealed by myocardial biopsy. None of the examined endomyocardial specimen revealed cardiac allograft rejection greater than Grade 1 R.

**Conclusion:**

A combined non-invasive approach using multi-slice computed tomography and magnetic resonance imaging may help to assess cardiac allograft vasculopathy and cardiac allograft rejection after heart transplantation before applying more invasive methods.

## Introduction

Since the development of heart transplantation for treatment of end-stage heart failure, the early diagnosis of transplant rejection has become essential. Regular follow-up contributes to detection of complications like coronary allograft vasculopathy and chronic transplant rejection which can result in significant graft coronary artery disease or myocardial fibrosis with loss of contractility. Coronary allograft vasculopathy is usually clinically silent and therefore presents a diagnostic challenge; because of continued denervation in the majority of heart transplants, any occurrence of myocardial ischemia in those grafts is asymptomatic with angina being rarely. They might manifest sequelae of coronary artery disease, including signs of congestive heart failure or loss of transplant function, or they may experience arrhythmias or sudden death.

Current diagnostic standards for the diagnosis of cardiac vasculopathy are invasive coronary angiography and for acute or chronic transplant rejection endomyocardial biopsy [1]. Endomyocardial biopsy is still the main technique for rejection surveillance. However biopsying is invasive and may be associated with complications [2]. Thus, there is a need for non-invasive methods. Non-invasive assessment of coronary vessels, left ventricular function and myocardial fibrosis has recently been examined by multi-slice spiral computed tomography and magnetic resonance imaging [3,4].

Our present pilot study was performed to analyze the relationship between the results from magnetic resonance imaging and those from endomyocardial biopsy respectively the end-points cardiac transplant rejection and its degree of severity. Further we conducted this study to investigate the relationship between the results from coronary angiography and those from the multi-slice spiral computed tomography respectively the end-points cardiac transplant vasculopathy and its degree of severity. The results should clarify if the non-invasive approach is reliable and could be superior in long time survivors after heart transplantation.

## Methods

### Routine follow-up for heart transplant patients

The routine follow-up consisted of clinical examination, blood testing, electrocardiogram and echocardiography and chest X-ray every three months.

### Blood testing

Routine blood testing consisted of red and white blood count with differential haemogram and cardiac enzymes (creatinin-kinase with its isoenzyme CK-MB and Troponine I). The parameters for liver (ALT, AST, ALP, GGT, LDH albumin and bilirubine), renal function (creatinine, urea) and coagulation (platelet count, INR and partial thromboplastin time) were part of the routine. Further serum analyses were lipids (triglycerides, HDL and LDL cholesterol), electrolytes (sodium, potassium, chloride and magnesium) and CRP. To rule out any infectious diseases virologic analyses with PCR were performed to detect cytomegaly virus, hepatitis A-E virus, herpes simplex, varicella zoster virus and human herpes virus 4. Finally the serum levels of the immunosuppressive drugs (cyclosporine A or mycophenolate) were analysed.

### Zytological analyses

This summarizes analyses of sputum, pharyngeal smear and urine to assess any bacterial, viral or fungal infections.

### Electrocardiogram

A 12-lead electrocardiogram was part of the basic follow-up.

### Echocardiography

To assess the patient's heart valves, ejection fraction and to rule out any vegetations and pericardial effusion patients were examined lying on their left side on the examination table. The images were displayed on a monitor and were recorded. Ultrasound device and probe (S5-1, 2.5 Mhz, iE33 Philips, Hamburg, Germany)

### Chest X-ray

Chest X-ray was routinely performed to evaluate the chest wall, lungs and heart.

### Transvenous endomyocardial biopsies

Transvenous endomyocardial biopsying was performed only as clinically indicated for systematic control or in case of suspected rejection. The systematic controls were performed at the following rates: at two-week intervals for the first four months following transplantation, then at monthly intervals until the end of the first year and finally at two-month intervals during the second year. After this period biopsying was performed in two year intervals.

### Study population and study protocol

10 patients (8 male, 2 female, mean age 52.1 ± 12 [29-64] years, mean time after heart transplantation 72.7 ± 11 months) were included in our study (Table 1). All patients gave informed consent before inclusion in the study. The study was approved by the hospital ethics committee. The study included besides one patient presenting with exercise induced dyspnea only asymptomatic patients without clinical and biochemical signs of an acute heart transplant rejection. Patients with an acute heart transplant rejection in the last three months were excluded. The body mass index was 26.8 ± 1.17 [22-34] kg/m^2^. 4/10 patients had an impaired renal function with serum urea levels between 50 and 100 mg/dl and serum creatinine levels between 1.2 and 1.7 mg/dl. 5/10 patients were still carrying out their professions on a daily base of 6-8 hours. The basic medication of all patients consisted of β-blockers, angiotensin converting enzyme inhibitors, statins, diuretics and the antiplatelet agent acetyl salicylic acid 100 mg per day. The immunosuppressive medication consisted in 8/10 patients of cyclosporin A and 2/10 patients received mycophenolate additionally. All patients were free of glucocorticoids.

**Table 1:** Patient characteristics and results.

**Patient**	**1**	**2**	**3**	**4**	5*	**6**	**7**	8*	**9**	10*
**Gender**	M	M	M	M	M	M	F	F	M	M

**Age [years]**	64	55	61	44	55	48	37	63	65	29

**BMI**	26	26	31	22	26	24	24	29	34	24

**Months after HTX**	89	67	70	63	79	81	90	69	62	68

**Prior rejections**	1	2	1	0	0	0	0	0	1	2

**CA**	NL	CAD	NL	NL	CS	NL	NL	NL	CS	CAD

**MSCT**	NL	CAD	NL	NL	CS	NL	NL	NL	CS	CAD

**Calcium mass**	0.06	0.13	0	0	0.81	0	0	0	11.3	NP

**Biopsy**	1 R	1 R	1 R	0	1 R	1 R	0	1 R	1 R	NP

**MRI**	DF	DF	DF	NF	NP	SF	NF	NP	DF	NP

**EF (%) by CA**	>55	<55	>55	>55	>55	>55	>55	>55	>55	>55

**EF (%) by MRI**	51	43	55	63	NP	60	75	NP	77	NP

**EF (%) by TTE**	61	48	57	71	61	61	73	62	70	69

M: male; F: female. (*) patient with cardiac pacemaker. BMI: body mass index in kg/m^2^. Calcium mass in mg CaHa. NL: no lesion. Biopsy: classification of rejection (ISHLT). Grade 0 R: no rejection; Grade 1 R: mild rejection; Grade 2 R: moderate rejection and Grade 3 R: severe rejection. Magnetic resonance imaging: NF: no fibrosis; SF: slight fibrosis; DF: diffuse fibrosis. NP: not performed. Coronary angiography/multi-slice computed tomography: CAD: Coronary artery disease; CS: Coronary sclerosis. Ejection fraction EF > 55% was defined as normal.

For the present study, which was undertaken to analyze the relationship between the results from magnetic resonance imaging and those from endomyocardial biopsy, magnetic resonance imaging investigations were retrospectively selected according to the following criteria: 1) a myocardial biopsy was obtained within one week of magnetic resonance imaging; 2) no intravenous treatment for acute rejection had been given in the week preceding magnetic resonance imaging or in the period between magnetic resonance imaging and myocardial biopsy; and 3) the patients were not identified as having a chronic transplant rejection at the time of these investigations.

Further in the present study the relationship between the results from coronary angiography and those from the multi-slice spiral computed tomography, multi-slice spiral computed tomography investigations were retrospectively selected according to the following criteria: 1) coronary angiography was performed within one week of multi-slice spiral computed tomography; 2) no intravenous treatment for acute rejection had been given in the week preceding multi-slice spiral computed tomography or in the period between multi-slice spiral computed tomography and coronary angiography; and 3) the patients were not identified as having a chronic transplant rejection at the time of these investigations.

Cardiologists performing the coronary angiography and endomyocardial biopsy, radiologists performing the multi-slice spiral computed tomography and magnetic resonance imaging and the pathologists performing the immunohistochemical analyses on the endomyocardial biopsies were blinded to the results of the different examinations.

### Coronary angiography

Standard (X-ray) coronary angiography was performed according to standard procedures. Stenosis severity was evaluated by quantitative coronary analysis (QCA, Philips, Eindthoven, Netherlands). Coronary angiography was performed in all patients.

Diffuse atherosclerosis was defined by wall irregularities in coronary angiography. Lesions with a diameter stenosis > 50% were considered to be significant and classified as a significant stenosis or coronary artery disease.

### Endomyocardial biopsying

Left ventricular endomyocardial biopsying (3 specimens per patient) could be performed in 9/10 patients. Endomyocardial biopsies were gained from three different regions of the left ventricular apex with a Cordis™ biotome with a jaw volume of 5.20 mm^3 ^under X-ray guidance. The obtained specimens were fixed in formaldehyde.

### Immunohistology

The fixed biopsies were embedded in paraffin, stained with Masson's trichrome and hematoxylin-eosin and examined by light microscopy. For immunohistological identification of cardiac immune cells 5 μm tissue sections were treated with avidin-biotin immunoperoxidase (Vectastain Elite ABC Kit, Vector, Burlingame, CA, USA), applying monoclonal antibodies: CD3 (T-cells, Novocastra Laboratories, Newcastle upon Tyne, UK), PGM1, HLA-DR (both DAKO, Hamburg, Germany). Graft rejection was classified according to the working formulation of the International Society for Heart and Lung Transplantation (ISHLT) [5]. In brief, the revised (R) categories of cellular rejection are as follows: Grade 0 R - no rejection; Grade 1 R - mild rejection; Grade 2 R - moderate rejection and Grade 3 R - severe rejection.

### Multi-slice spiral computed tomography

Multi-slice spiral computed tomography was performed by using a Sensation 16 Speed 4 D™ (Siemens Medical Solutions, Forchheim, Germany) scanner. This technique allows the application of dedicated spiral algorithms that provide up to 185 ms of temporal resolution. electrocardiogram-gated heart phase selective imaging reconstruction was used in all patients. As all our heart transplant patients received β-blockers, no further β-blockade prior to the multi-slice spiral computed tomography scan was performed. After a low dose precontrast spiral scan (collimation 0.75 mm, 2.8 mm table feed/rotation, 120 kV, 133 mAs, rotation time 370 ms) with simultaneously recorded electrocardiogram signal, a test bolus of 20 ml of contrast medium and a chaser bolus of 20 ml of physiological saline solution were injected through an 18-gauge catheter into an antecubital vein to determine the circulation time. The following scan protocol was used: 0.75 mm collimation, caudocranial scan direction, 80 cc contrast media (400 mg Iodine/cc) with a biphasic injection protocol (50 ml at 4 ml/s and 30 ml at 2.5 ml/s), gantry rotation time 370 ms, temporal resolution 185 ms, effective slice thickness 1.0 mm, 120 kV, approximately 650 mAs. All scans could be performed within one single breath-hold (15-20 s). Algorithms optimized for retrospective electrocardiogram-gated multi-slice spiral computed tomography were used for reconstructing the raw data. Image reconstruction was performed in the diastolic phase with a relative retrospective gating between 20% and 75% of the RR-interval. Multi-slice spiral computed tomography radiation dose was approximately 6 mSv.

The reconstructed data of the multi-slice spiral computed tomography were transferred to a computer workstation for further processing (Leonardo™, Siemens Medical Solutions, Forchheim, Germany)

### Multi-slice spiral computed tomography image interpretation

The scans were evaluated by two independent radiologists blinded to clinical data coronary angiography and biopsy results in a joint reading. Data were analysed on an offline workstation for postprocessing (Leonardo™, Siemens, Forchheim, Germany). Coronary calcifications were assessed on native scans and quantified by determining the total calcium mass expressed in mg of calcium hydroxyapatite (CaHa). Morphological changes with resulting narrowing of the coronary artery diameter served as another criterion to assess coronary artery disease. Lesions with a diameter stenosis > 50% were considered to be significant and classified as a significant stenosis or coronary artery disease.

### Magnetic resonance imaging

Magnetic resonance imaging could only performed in 7/10 patients includes into this study. 3 patients were excluded due to contraindications (implanted pacemakers). Total measurement time for magnetic resonance examinations was within 45 minutes in all patients. Electrocardiogram-triggered cardiac magnetic resonance examinations were performed on a 1.5T MR scanner (Magnetom Sonata™, Siemens Medical Solutions, Forchheim, Germany). Cine images (repetition time 3.08 ms, echo time 1.54 ms, flip angle 50°, temporal resolution 46 ms), T1 weighted turbo spin echo images (repetition time 700 ms, echo time 24 ms, flip angle 180°, matrix 125 × 256, band width 305 Hz/Pixel) and T2 weighted turbo spin echo images (repetition time 1800 ms, echo time 84 ms, flip angle 180°, matrix 160 × 256, band width 235 Hz/Pixel), as well as delayed enhancement images using an inversion recovery-TurboFLASH sequence (repetition time 9.56 ms, echo time 23 ms, inversion time 200-260 ms, flip angle 25°, matrix 166 × 256) were acquired in the three main cardiac axes. Cine short axis sections were recorded from base to apex for subsequent functional evaluation. For post contrast/delayed enhancement images there was a delay of 15 minutes between injection of 0.15 mmol Gadolinium-DTPA/kg body weight (Magnevist™, Schering AG, Berlin, Germany) and image acquisition. Inversion time was adjusted in order to minimize signal of normal myocardium.

### Magnetic resonance image interpretation

The scans were evaluated by two independent radiologists blinded to clinical data, multi-slice computed tomography, coronary angiography and biopsy results in a joint reading. Electrocardiogram-triggered cardiac magnetic resonance imaging examinations were performed on a 1.5 Tesla magnetic resonance scanner (Magnetom Sonata™, Siemens Medical Solutions, Forchheim, Germany). Ejection fraction was calculated using short axis cine images. T1 weighted images, T2 weighted images and late contrast enhancement images were assessed later [6,7]. The focus of interest was late enhancement especially in the left ventricle. Location of pathologic signal enhancement was classified as ‚local' or ‚diffuse', whereas the severity was rated on a 3-point scale: weak, moderate, and severe signal enhancement.

### Statistics

Continuous variables are described as means and standard error of mean. We used Prism 5.0™ (GraphPad Software Inc., San Diego, CA, USA) for the analyses. Comparisons between discrete variables and comparisons between proportions were made by calculating the Pearson product-moment correlation coefficient. For all tests, a p value ≤ 0.05 was considered to be indicative of a significant difference.

## Results

### Comparison of coronary angiography and multi-slice spiral computed tomography

**Figure 1:**
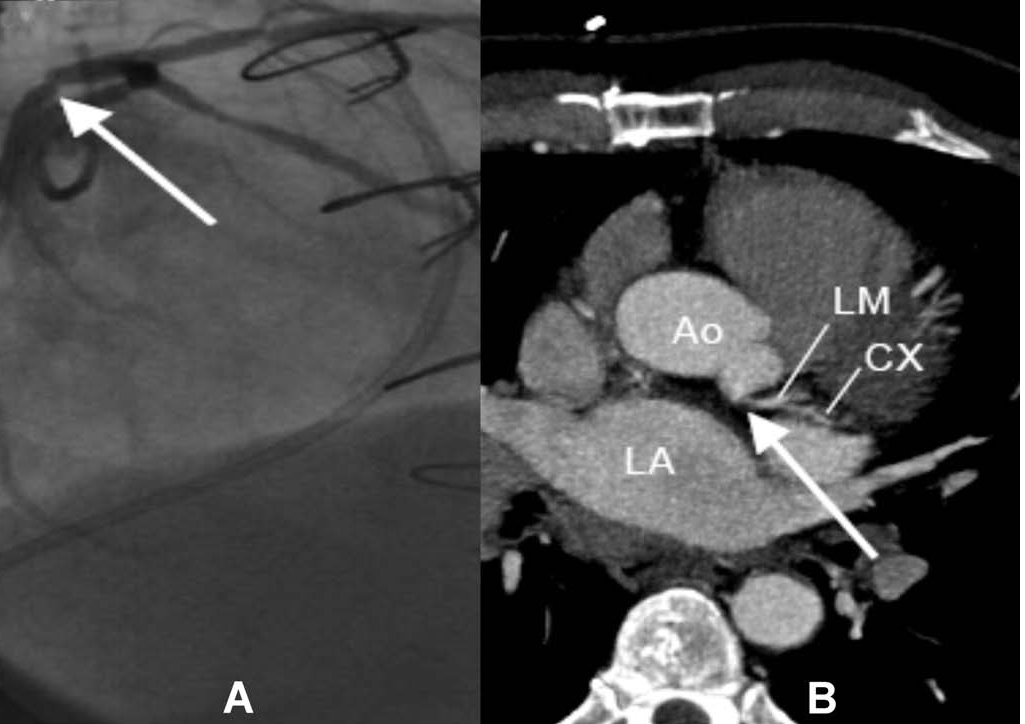
**A: Coronary angiogram showing the stenosis of the left main stem in patient number 2 (arrow, LAO: left anterior oblique)**. B: Multi-slice computed tomography of the same patient with the corresponding stenosis (arrow, MIP: maximal intensity projection). Ao: aorta, LA: left atrium, LM: left main artery, CX: circumflex artery.

By coronary angiography and multi-slice spiral computed tomography lesions in the epimyocardial vessel segments could be correctly detected in all patients (Table 1). 2 patients showed diffuse atherosclerotic lesions (patient no. 5 Calcium mass 0.81 in mg CaHa, patient no. 9 Calcium mass 11.3 in mg CaHa) and coronary artery disease was diagnosed in 2 patients (patient no. 2: stenosis of the left main stem and left anterior descending artery (Figure 1), Calcium mass 0.13 in mg CaHa; patient no. 10: stenosis of the left anterior descending artery which was treated 5 days later by percutaneous coronary intervention. Patient no. 2 was meanwhile also treated by percutaneous coronary intervention. The complications after coronary angiography were haematoma of the groin in 2 patients and pericardial effusion in 2 patients, which could be managed conservatively. After multi-slice spiral computed tomography no complications occurred.

The results of coronary angiography and multi-slice spiral computed tomography correlated positively with a Pearson coefficient r = 1.

No significant correlation could be demonstrated between the Calcium mass and the degree of severity of the coronary lesion (r = 0.32, r^2 ^= 0.1, p = 0.4).

### Comparison of magnetic resonance imaging and endomyocardial biopsy

Late contrast enhancement as a sign of myocardial injury or scarring after prior rejections [6,7] was considered as fibrosis and was found in 4 patients (no. 1, 2, 3 and 9). Severely reduced left ventricular contractility was found in patient no. 2 with coronary artery disease and two prior acute rejections.

**Figure 2:**
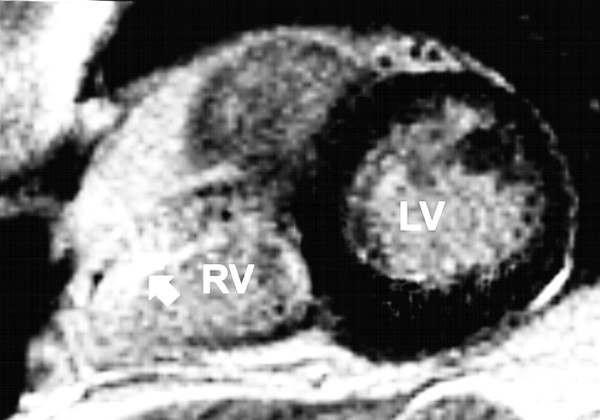
**Magnetic resonance imaging of patient number 4 (short axis) featuring a homogenous pattern of the left ventricular myocardium (late enhancement, segmental inversion recovery - TurboFLASH 2D image)**. RV: right ventricle. LV: left ventricle. Arrow marks right ventricular late enhancement.

**Figure 3:**
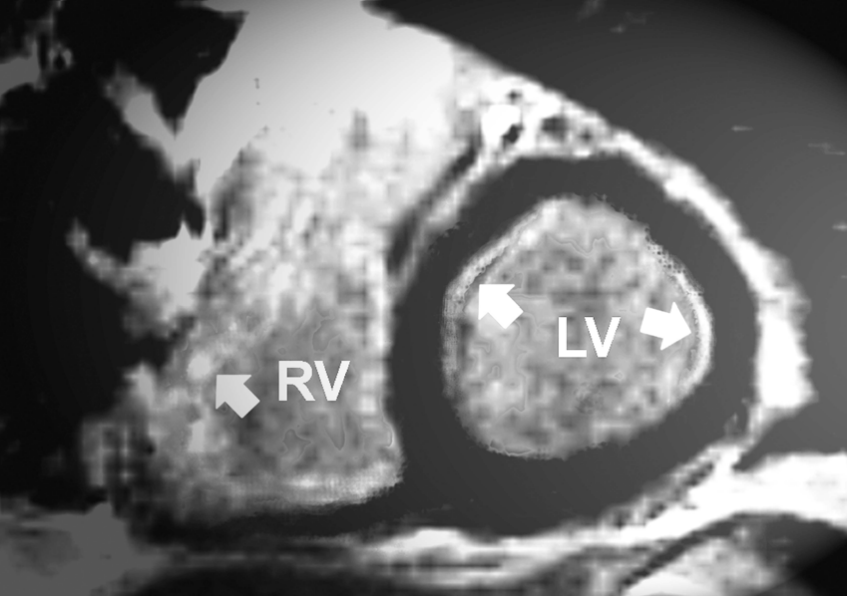
**Magnetic resonance imaging of patient number 6 (short axis) showing a signal enhancement of the septal (arrow) and slightly of the basolateral region (arrow) of the left ventricular myocardium (late enhancement, segmental inversion recovery - TurboFLASH 2D image)**. RV: right ventricle. LV: left ventricle.

**Figure 4:**
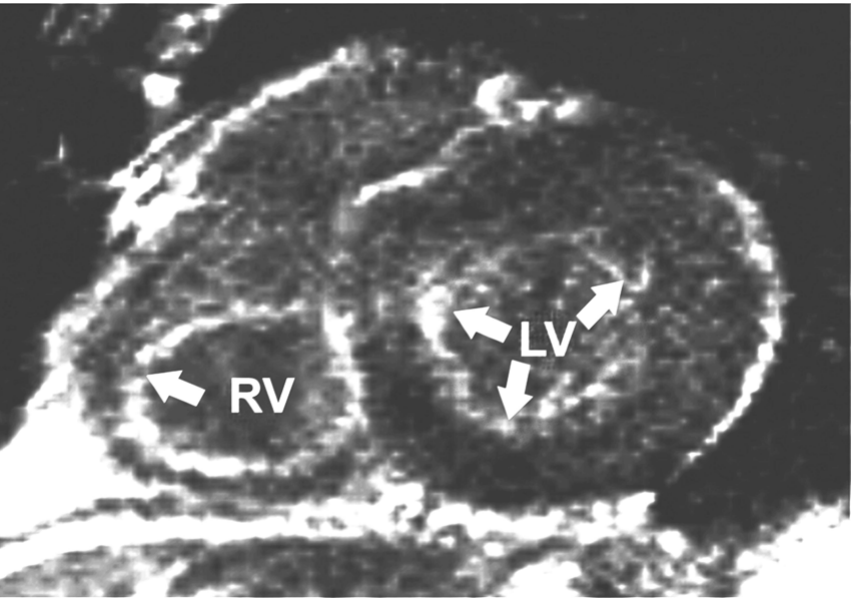
**Magnetic resonance imaging of patient number 1 (short axis) showing a diffuse signal enhancement (arrows) of the left ventricular myocardium (late enhancement, segmental inversion recovery - TurboFLASH 2D image)**. RV: right ventricle. LV: left ventricle.

We found three different pattern of late contrast enhancement in the left ventricle (Table 1): none in patient no. 4 (Figure 2) and 7, slight or focal late contrast enhancement in patient no. 6 (Figure 3) and diffuse late contrast enhancement in patient no. 1 (Figure 4), 2, 3 and 9.

The endomyocardial biopsies of patient no 4 and 7 did not feature a rejection. In contrast to that in the endomyocardial biopsies of 7 patients (patient no. 1, 2, 3, 5, 6, 8 and 9) mild transplant rejection (Grade 1 R) was evident. In none of the patients a rejection greater than Grade 1 R existed

There was a significant correlation between the results of the magnetic resonance imaging late left ventricular enhancement sequences and the left ventricular endomyocardial biopsies (p < 0.05). Furthermore, the endomyocardial biopsy results respectively the histologically determined degree of rejection correlated positively with the late left ventricular enhancement confirming fibrosis (r = 0.92, r^2 ^= 0.85, p < 0.05).

Another significant correlation existed between the results of the magnetic resonance imaging late left ventricular enhancement sequences and the number of prior acute transplant rejections (r = 0.83, r^2 ^= 0.69, p < 0.05).

## Discussion

Early detection of acute heart transplant rejection is important, as immediate treatment contributes to a lower incidence of rejection complications. The diagnostic gold standard is still an endomyocardial biopsy with additional staining for CD3 and HLA-DR positive cells. Biopsying, however, carries considerable risks [8]. Therefore non-invasive methods like magnetic resonance imaging and multi-slice spiral computed tomography could be beneficial. As a late complication after heart transplantation allograft vasculopathy should be recognized also as early as possible to prevent a worse outcome. If considering that the diagnostic standard for the detection of transplant vasculopathy is still coronary angiography with intracoronary ultrasound the demand for a non-invasive assessment like presented in our current study exists. In our study we could demonstrate that by utilizing multi-slice spiral computed tomography stenosis or arteriosclerotic lesions in the epimyocardial vessel segments as seen in coronary angiography just in accordance to previous studies [9] could be detected reliably. Future improvements of the resolution capacity of multi-slice spiral computed tomography could allow for assessing vasculopathy in even much smaller vessel segments. The radiation exposure caused by multi-slice spiral computed tomography and coronary angiography was almost equal. During their clinical course patients after heart transplantation are exposed to a high radiation dose due to many chest X-rays and coronary angiography. Thus additional radiation exposure should be minimized to avoid radiation associated risk of cancer. Furthermore, for multi-slice spiral computed tomography and coronary angiography the use of iodinated contrast media is necessary which could add to existing impaired renal function. In mid-term follow-up no significant changes in renal or thyroid function after contrast media exposure occurred at our patients.

Multi-slice computed tomography combines the advantages of angiography for lumen imaging and of intravascular ultrasound for coronary wall imaging, and it may have the potential to surpass coronary angiography in the diagnosis of coronary allograft vasculopathy [10]. Multi-slice computed tomography as a non-invasive application warrants its superiority over coronary angiography just like presented in our study in the lack of any complications. In comparison to that 4 patients featured complications after coronary angiography with resulting longer hospital stay. Like presented in our study utilizing a 16-channel multidetector computed tomography scanner to evaluate the utility of computed tomography for the detection of coronary allograft vasculopathy, Romeo et al reported a sensitivity of 83%, specificity of 95% for the detection of coronary artery stenoses greater than 50% in a prospective 53-patient series [11]. In comparison to these results in our present pilot study a positive correlation existed between the findings of the computed tomography and coronary angiography. Lesions in the coronary arteries could be ruled out or detected correctly each time. No significant correlation could be demonstrated between the calcium mass and the degree of severity of the coronary lesion. One explanation could be the different pathogenesis of coronary lesions in cardiac allograft vasculopathy [12].

### Cardiac allograft vasculopathy

Cardiac allograft vasculopathy is a unique form of atherosclerosis that results from chronic immunemediated injury to the transplanted heart, combined with multiple nonimmunologic factors and therefore is distinct from coronary artery disease acquired due to atherosclerosis [12]. Coronary allograft vasculopathy causes endothelial damage, which often results in luminal narrowing, myocardial ischemia, and ultimately graft failure [13]. The estimated risk for the development of coronary allograft vasculopathy in heart transplant recipients is 10% per year. The disease process in coronary allograft vasculopathy differs from that in classic atherosclerosis both anatomically and histologically [13,14]. Luminal narrowing typically begins in the distal small coronary arteries and progresses proximally to the epicardial vessels. Collateral vessels are remarkably absent. Pathologically, there is diffuse concentric atherosclerotic narrowing rather than the focal, patchy, and often eccentric disease that typifies classic atherosclerosis, in which collateral vessels are common [13]. For multi-slice spiral computed tomography diagnosis of atherosclerosis coronary calcifications were assessed visually, and they were quantitatively determined, based on the standard built-in algorithm using an adapted Agatston-score equivalent [15]. This scoring system, however, has a limited reproducibility [16-18]. Therefore we measured the total calcium mass expressed in mg of calcium hydroxyapatite (CaHa). Coronary sclerosis and intimal wall thickening with at least 50% reduction of the vessel diameter was classified as a significant stenosis or coronary artery disease. The use of calcium mass as a quantitative index for the amount of calcium is more precise than are other methods because the pixels that compose each calcified lesion are corrected by an appropriate calibration factor to compensate for the decreased mean computertomogram numbers that result from the linear partial volume effects [19].

Coronary allograft vasculopathy is usually clinically silent without angina due to denervation in the majority of cardiac allografts [20]. Patients might manifest sequelae of coronary artery disease, including signs of congestive heart failure or loss of allograft function [21]. Early diagnosis of cardiac allograft vasculopathy is important because the prevention of impending catastrophic events is feasible in some patients through revascularization - either percutaneously with balloon angioplasty and with or without stent implantation, or by means of bypass surgery. Like presented above in our study coronary angiography and multi-slice spiral computed tomography could reveal a significant coronary disease interpreted as cardiac allograft disease in two patients. Except for one of the patients with exercise induced dsypnea no other clinical signs existed. Both patients were treated within one week percutaneously with balloon angioplasty and stent implantation. Thus in our present study multi-slice spiral computed tomography proved a useful non-invasive tool in the assessment of transplant vasculopathy. Despite the coincidental diagnosis of coronary sclerosis or intimal wall thickening in two patients and coronary artery disease in further two patients we could not demonstrate a positive correlation between the measured Calcium mass and the detected coronary lesions. Therefore morphological changes with resulting narrowing of the coronary artery diameter served as another criterion to assess coronary artery disease.

### Cardiac allograft rejection

The ability of magnetic resonance imaging to characterize ventricular morphology, systolic function, diastolic function, and myocardial inflammation makes it an excellent candidate to noninvasively diagnose and screen for heart transplant rejection unaware of its degree of severity. Normal myocardium does not show late contrast enhancement because Gadolinium(III)-diethyltriaminepentaacetic acid (Gd-DPTA) does not accumulate in the intracellular or interstitial space. Gd-DPTA accumulation may reflect an increase of interstitial space of inflammatory and fibrotic tissue as well as different wash-out kinetics in those areas. Gd-DPTA late enhancement is a tissue- or necrosisspecific staining technique or a ligand binding to specific receptors [6,7,22]. Gadolinium is an inert extracellular contrast agent and the amount of contrast agent in a given tissue distribution volume determines the image signal intensity - the more contrast per distribution volume the higher the signal. An important physiological fact to remember is that the tissue volume in normal myocardium is predominately intracellular (~75% of the water space). Because extracellular contrast media is excluded from this space by the intact sarcolemmal membrane, the volume of distribution of a contrast medium in normal myocardium is quite small (~25% of water space), and one can consider viable myocytes as actively excluding contrast media. The unifying mechanism for the hyperenhancement effect of nonviable myocardium may then be the absence of viable myocytes rather than any inherent properties that are specific for acutely necrotic tissue, collagenous scar, or other forms of nonviable tissue [23-25]. Thus there are two possible mechanisms for a signal enhancement in cardiac allograft rejection- fibrosis and inflammation [7]. It is well described that cardiac allograft rejection does not show a uniform distribution and that recurrent right ventricular endomyocardial biopsying may result in local scars, as existed in all of our patients included in the present study. Therefore we gained left ventricular endomyocardial biospies. In a former study histological examinations revealed no significant difference between right and left ventricular rejection [26]. A previous study was able to disclose myocardial fibrosis already in patients with absent or mild angiographic cardiac allograft vasculopathy [27]. This could be a useful diagnostic tool for the detection of earlier cardiac allograft vasculopathy and enable an intensified medical treatment.

### Limitations of this study

Our current study is a pilot study including only 10 patients with a mean time after heart transplantation of 72.7 ± 11 months. In these long-term survivors after heart transplantation acute cardiac allograft rejections according Grade 2 R and higher are not frequent as in the first years after transplantation. We could only detect mild chronic transplant rejection in our study population equivalent to Grade 1 R (ISHLT). Patients with cardiac pacemakers could not be evaluated by magnetic resonance imaging.

## Conclusion

In this pilot study multi-slice spiral computed tomography proved to be equal to coronary angiography to detect cardiac allograft vasculopathy in epimyocardial vessel segments in patients after heart transplantation. The results of multi-slice spiral computed tomography and coronary angiography showed a high positive correlation. In regard of complications multi-slice spiral computed tomography proved superior to coronary angiography enabling it without any hospital stay underlining its cost-effectiveness.

Magnetic resonance imaging revealed left ventricular fibrosis which seems to correlate with the histological findings of the endomyocardial biopsy showing a mild cardiac allograft rejection Grade 1 R according to the ISHLT working formulation.

A combined non-invasive approach seems to be useful, cost-effective and less harmful for the patient for detection of cardiac allograft vasculopathy and cardiac transplant rejection before applying invasive methods. Larger studies should be performed to improve the sensitivity detecting cardiac allograft rejection and possibly reduce, if not eliminate, the need for endomyocardial biopsy especially in patients with acute and severe cardiac allograft rejection with at least Grade 2 R.

## Competing interests

The authors declare that they have no competing interests.

## Authors' contributions

EU carried out the routine follow-up examinations, echocardiographies and participated in the study design and coordination. EU performed the statistical analysis. CB, SS and UH carried out the echocardiographies, coronary angiographies and endomyocardial biopsying. Andreas F K carried out the multi-slice computed tomography and magnetic resonance imaging and participated in their analyses. HA and GZ conceived of the study, and participated in its design and coordination. All authors read and approved the final manuscript.
